# Accounting for Genetic Differences Among Unknown Parents in *Bubalus bubalis*: A Case Study From the Italian Mediterranean Buffalo

**DOI:** 10.3389/fgene.2021.625335

**Published:** 2021-02-04

**Authors:** Mayra Gómez, Dario Rossi, Roberta Cimmino, Gianluigi Zullo, Yuri Gombia, Damiano Altieri, Rossella Di Palo, Stefano Biffani

**Affiliations:** ^1^Italian National Association of Buffalo Breeders, Caserta, Italy; ^2^Department of Veterinary Medicine and Animal Production, University of Federico II, Naples, Italy; ^3^Institute of Agricultural Biology and Biotechnology, National Research Council, Milan, Italy

**Keywords:** buffalo, breeding values, unknown parent groups, type traits, heritability

## Abstract

The use of genetic evaluations in the Water Buffalo by means of a Best Linear Unbiased Prediction (BLUP) animal model has been increased over the last two-decades across several countries. However, natural mating is still a common reproductive strategy that can increase the proportion of missing pedigree information. The inclusion of genetic groups in variance component (VC) and breeding value (EBV) estimation is a possible solution. The aim of this study was to evaluate two different genetic grouping strategies and their effects on VC and EBV for composite (*n* = 5) and linear (*n* = 10) type traits in the Italian Mediterranean Buffalo (IMB) population. Type traits data from 7,714 buffalo cows plus a pedigree file including 18,831 individuals were provided by the Italian National Association of Buffalo Breeders. VCs and EBVs were estimated for each trait fitting a single-trait animal model and using the official DNA-verified pedigree. Successively, EBVs were re-estimated using modified pedigrees with two different proportion of missing genealogies (30 or 60% of buffalo with records), and two different grouping strategies, year of birth (Y30/Y60) or genetic clustering (GC30, GC60). The different set of VCs, estimated EBVs and their standard errors were compared with the results obtained using the original pedigree. Results were also compared in terms of efficiency of selection. Differences among VCs varied according to the trait and the scenario considered. The largest effect was observed for two traits, udder teat and body depth in the GC60 genetic cluster, whose heritability decreased by −0.07 and increased by +0.04, respectively. Considering buffalo cows with record, the average correlation across traits between official EBVs and EBVs from different scenarios was 0.91, 0.88, 0.84, and 0.79 for Y30, CG30, Y60, and CG60, respectively. In bulls the correlations between EBVs ranged from 0.90 for fore udder attachment and udder depth to 0.96 for stature and body length in the GC30 scenario and from 0.75 for udder depth to 0.90 for stature in the GC60 scenario. When a variable proportion of missing pedigree is present using the appropriate strategy to define genetic groups and including them in VC and EBV is a worth-while and low-demanding solution.

## Introduction

The Water Buffalo (*Bubalus bubalis*) is a large bovid mainly distributed in the Asian continent where the 97% of its world population is concentrated [Food and Agriculture Organization (FAO), 2020]. The name “water buffalo” is due to its adaptation to flooded or swampy areas, where it partially submerges and walks on the bottom mud without difficulty. The rest of the water buffalo world population (3%) is raised in the Mediterranean area historically characterized by the same optimal rearing conditions. In the European continent only the 0.2% of its world population is found and about 93% of these animals are located in south-central Italy (Neglia et al., 2020). The total census in Italy has increased considerably over the last decade, making it one of the most important dairy species in the country. In 2019, 34,990 lactating buffaloes have been registered to the official herd book. Moreover, 666,960 controlled lactations and 9,953 type traits evaluations are available and officially recorded [Associazione Nazionale Allevatori Specie Bufalina (ANASB), 2020]. Thanks to the physical-chemical properties of its milk—high concentration in protein and fat (FC ∼ 8%) and favorable coagulation (Costa et al., 2020b)—the main zootechnical interest of the Italian Mediterranean Buffalo (IMB) is the production of the iconic traditional dairy products like the Mozzarella di Bufala Campana (Boselli et al., 2020), which has a great economic impact on the Italian food industry (ISMEA, 2020). Costa et al. (2020a, b) refers to the outstanding increase of IMB population size observed in the last 15 years, as well as the increase in terms of kilos of cheese produced, the larger herd size, the constant expansion in registered herds and the increment in milk price. Therefore, the economic interest in this specie makes it necessary to develop new innovative tools to improve the breeding process.

The implementation of genetic evaluations in the Water Buffalo based on a BLUP animal model has been increasing over the last decade across several countries (Agudelo-Gómez et al., 2015; Safari et al., 2018; Abdel-Shafy et al., 2020). The prediction of breeding values (EBVs) constitutes an integral part of most breeding programs which are based on two fundamental pillars: phenotypic data (e.g., milk production%, fat%, protein, or morphological trait) and genealogical information (i.e., a pedigree). However, if animals with unknown parents are present in the pedigree, bias in the prediction of both variance component (VC) and EBV is expected (Peškovičová et al., 2004; Petrini et al., 2015). BLUP methodology allows for the simultaneous estimation of fixed and random effects but gaps in the relationship matrix may jeopardize its unbiasedness due to the inability of correctly estimating and disentangling genetic and environmental components (Postma, 2006; Gómez et al., 2016; Wolak and Reid, 2017). Indeed, incomplete pedigree information can lead to inaccurate prediction of animal genetic potential, overestimating or underestimating animal breeding value and hampering decisions based on the selection eventually causing economic losses (Raoul et al., 2016; Carneiro et al., 2017; Abdel-Shafy et al., 2020).

One of the reason behind incomplete pedigree information is the use of natural mating, still common in the buffalo herds, which makes parentage assignment more complex. Indeed, in IMB the use of the artificial insemination (AI) is still moderate (Parlato and Van Vleck, 2012). According to official data [Associazione Nazionale Allevatori Specie Bufalina (ANASB), 2020] and following a worldwide tendency (Singh and Balhara, 2016; Purohit et al., 2019), the proportion of natural mating in IMB decreased from around 76 to 62% from 2010 to 2019 [Associazione Nazionale Allevatori Specie Bufalina (ANASB), 2020]. These values, even if promising, are still lower than what it is observed in other species such as in dairy cattle, where the use of artificial insemination is close to 100% (Rodríguez-Martínez and Peña Vega, 2013; Ugur et al., 2019). Among the reasons why natural mating is still the most common reproduction technology for water buffalo there are physiological and reproductive aspects, herd management, breeding techniques, and organization (Neglia et al., 2020).

Despite being a routine analysis, it is almost impossible for the farmer to bear the total cost of parentage verification and to have his entire herd genotyped. In detail, in 2019 approximately 10,000 individuals have received a type trait evaluation in Italy but only 4,671 were DNA tested [Associazione Nazionale Allevatori Specie Bufalina (ANASB), 2020]. Hence, we are in a situation where phenotypic data are available for many animals, but a large proportion of these animals do not have complete pedigree information. Despite this limitation, the number of paternity tests in IMB in year 2019 showed a two-fold increase compared to year 2018.

Moreover, parentage testing is often reserved only for the best animals causing additional biases in the genetic evaluation being eventually based on a selected and non-random sample of the effective population. Furthermore, the possibility of using a larger number of data, albeit with incomplete pedigree, allows to observe all the variability of the trait of interest and therefore to obtain more accurate estimates.

The problem of incomplete pedigree has existed for many years and continues to be one of the main issues in genetic evaluations. Several researchers have worked on possible statistical approaches in order to correct for the presence of gaps in the pedigree (Peškovičová et al., 2004; Carneiro et al., 2017; Tonussi et al., 2017; Shiotsuki et al., 2018; Nwogwugwu et al., 2020; Macedo et al., 2020). The implementation of new technologies such as high-throughput single-nucleotide polymorphism (SNP) genotyping will certainly solve most of the problems linked to uncertain paternity but this is true only for individuals who are still alive or whose biological samples are available. Moreover, although genomics is the new standard in breeding and genetics, there are still some problems that need to be solved regarding how to cope with missing pedigree information (Tonussi et al., 2017; Misztal et al., 2020).

One suggested solution when dealing with an incomplete pedigree is the use of “Genetic Groups” approach, suggested over 30 years ago by Westell et al. (1988). This approach is based on the concept that subjects born in a certain period or coming from a certain area are the result of specific selective choices and therefore “genetically different” from other subjects born in other periods or from other areas.

The inclusion of genetic groups in VC and EBV is a method that has been adopted and extensively validated, as an example, in beef and dairy cattle (Perez-Enciso and Fernando, 1992; Sullivan, 1995; Theron et al., 2002; Peškovičová et al., 2004; Phocas and Laloë, 2004; Petrini et al., 2015; Wolak and Reid, 2017). The assignment of genetics groups to animals with uncertain genealogy represents a simple and effective solution to increase the accuracy of genetic evaluations (Henderson, 1988; Cardoso and Tempelman, 2003).

However, a crucial aspect is the strategy used to define the genetic groups. Therefore, the aim of this study was to evaluate the use of different genetic grouping strategies and its effects on VC and EBV estimation for 5 composite and 10 linear traits in the IMB population.

## Materials and Methods

### Ethics Statement

Animal welfare and use committee approval was not needed for this study as datasets were obtained from pre-existing databases based on routine animal recording procedures.

### Data Description

Data for the present study were provided by the Italian National Association of Buffalo Breeders (ANASB) and consisted of linear appraisal records from years 2004 to 2020. The initial data set included 79,342 IMB cows from 464 herds phenotyped for fifteen type traits. The type traits were five composite traits, namely, final score (FS), structure (ST), feet and legs (FL), yield potential (YP) and udder teat (UT), and 10 linear traits, namely, stature (STAT), body depth (BD), body length (BL), foot angle (FA), fore udder attachment (FUA), rear udder width (RUW), udder depth (UD), teat placement (TP), teat length (TL), and body condition score (BCS). The median age at evaluation was 46 months. The scale used for scoring varied according to the set of observed traits. Composite traits were scored on a 65–100 scale, linear traits were scored on a 1–50 scale and BCS was scored on a 4.5–9.5 scale. Overall 17 official classifiers were enrolled in the scoring procedures. Data editing consisted of retaining only cows from herds with at least two contemporaries (i.e., individuals classified by the same classifier in the same round of classification) and whose ascendants were confirmed by a DNA parentage test. Finally, 7,714 buffalo cows belonging to 194 herd with a pedigree containing 18,831 individuals were used in the analysis. Descriptive statistics are in Table 1.

**TABLE 1:** Mean, standard deviation (SD), minimum (Min), maximum (Max), and coefficient of variation (CV) for traits evaluated in the IMB.

Type	Trait	Mean	SD	Min	Max	CV
Composite	Final score (FS)	81.34	1.82	65	87	0.02
	Structure (ST)	82.50	2.38	69	91	0.03
	Feet and legs (FL)	80.19	2.59	65	89	0.03
	Under teat (UT)	80.30	2.64	65	90	0.03
	Yield potential (YP)	83.44	2.14	71	90	0.03
Linear	Stature (STAT)	30.57	6.56	8	50	0.21
	Body depth (BD)	29.48	6.00	7	50	0.20
	Body length (BL)	31.50	6.56	10	50	0.21
	Foot angle (FA)	22.65	6.14	3	50	0.27
	Fore udder attachment (FUA)	22.39	6.84	2	46	0.31
	Rear udder width (RUW)	24.20	6.12	2	50	0.25
	Udder depth (UD)	27.69	6.33	2	50	0.23
	Teat placement (TP)	21.30	4.74	1	50	0.22
	Teat length (TL)	23.85	7.04	2	50	0.30
	Body condition score (BCS)	7.34	0.47	4.5	9.5	0.06

### Alteration of Genetic Relationships and Grouping Strategies

The impact of different genetic grouping strategies on VC, EBV, and their accuracies (ACC) was investigated using the original pedigree and a modified pedigree where two different proportion of missing genealogies, namely, 30% (30) and 60%, (60) were randomly introduced. The choice of using these two thresholds was based not only on the need to mimic the real situation observed across ANASB farms but also to investigate the effect of moderate or massive pedigree gaps. After introducing the missing genealogy, the individual was assigned to a specific genetic group. Genetic groups (GG) were created following two clustering methods.

The first method (Y) was based on the year of birth and on an average generation interval, which for the IMB was defined (based on an estimation on actual IMB data) as 6 years. Individuals born before 1985 was considered as base animals and assigned to group 1. The remainder of the buffaloes was assigned to six different groups.

The second grouping strategy (GC) was based on the genetic distances estimated from the original pedigree. The procedure consisted of two steps. In the first step the pedigree-based additive relationship matrix was calculated and used as input for a hierarchical cluster analysis using a complete-linkage clustering method (Kaufman and Rousseeuw, 2009). This method works in a bottom-up manner. Each object is initially considered as a single-element cluster (leaf). At each step of the algorithm, the two clusters that are the most similar are combined into a new bigger cluster (nodes).

This procedure is iterated until all points are member of just one single big cluster (root). The result is a tree that can be plotted as a dendrogram. In the second step, the dendrogram is visually evaluated to define *a priori* the cut-off level that will identify the number of clusters (i.e., genetic groups). Each individual is then assigned to a particular cluster. Following the above mentioned procedure, fourteen different genetic groups were created (Supplementary Figure 1).

In detail at the end of the procedures, four scenarios were created according to the grouping strategy (Y or GC) and the proportion of missing genealogies (30 or 60%).

Successively, VC, EBV, and ACC were estimated for each trait presented in Table 1 fitting a single-trait animal model and using the original pedigree (GOLD) and the four scenarios, namely Y30, Y60, GC30, and GC60. Estimates from GOLD were considered as *gold standard*. The estimation of VC, EBV, and ACC was repeated 10 times per each scenario (Y30, Y60, GC30, and GC60). The average number of animals and its standard deviation per scenario are shown in Table 2.

**TABLE 2:** Average number of animals (and standard deviation) by genetic grouping strategy (GG) and proportion of missing genealogies.

GG	Level	Proportion of missing genealogies	
		30%	60%
Y^a^	1	43 (0)	43 (0)
	2	456 (1)	456 (1)
	3	1,798(87)	1,800(89)
	4	2,524(432)	2694 (607)
	5	2,394(412)	2,876(906)
	6	1,001(271)	1,435(715)
	7	148 (35)	218 (106)
GC^b^	1	5,973(656)	6,279(656)
	2	695 (52)	985 (54)
	3	369 (22)	559 (23)
	4	279 (53)	450 (55)
	5	291 (81)	468 (81)
	6	345 (96)	556 (96)
	7	134 (35)	218 (35)
	8	356 (90)	579 (90)
	9	206 (58)	330 (57)
	10	101 (31)	162 (30)
	11	219 (61)	353 (61)
	12	238 (56)	393 (56)
	13	249 (65)	397 (66)
	14	69 (21)	109 (22)

*^a^Grouping strategy based on the year of birth and on an average generation interval set to 6 years. ^b^Grouping strategy based on the genetic distances estimated from the original pedigree.*

### Genetic Analysis

The following single-trait animal model with groups was used to estimate VC, their corresponding heritability, and breeding value for each considered trait:

$$\begin{array}{c} y_{ijklm}=\mu +hyc_{i}+PA_{j}+DIM_{k}+NM_{l}+a_{m}\\ \;+\sum_{n=1}^{p}t_{mn}g_{n}+e_{ijklm} \end{array}$$

where *y*_*ijklm*_ is the score of each trait for a given buffalo cow; μ is the overall mean; hyc_i_ is the fixed effect of the *i*th herd-year of evaluation-classifier (*i* = 1,…957); *PA*_*j*_ is the fixed effect of the *j*th age nested within parity (*j* = 1,…173); *DIM*_*k*_ is the fixed effect of the *k*th days in milk (*k* = 1,…30); *NM*_*l*_ is the fixed effect of the *l*th number of milking (*l* = 1,…3); *a*_*m*_ is the random additive genetic effect of the *m*th buffalo; *g*_*n*_ is the fixed group effect based on Y or GG and containing the *n*th ancestor; *t*_*mn*_ is the additive relationship between the *n*th and *m*th animals and the summation is over all *p* ancestors of animal *m*; and *e*_*ijklm*_ is the random residual effect.

In matrix notation, the model can be written as:

$$y=Xb+Z_{a}Q_{a}g_{a}+Z_{a}a+e$$

*w*here matrix *X* is an incidence matrix relating phenotypic records in vector *y* to fixed effects in vector *b*, matrix *Z*_*a*_ is an incidence matrix relating phenotypic records in vector *y* to animal additive genetic effects in vector *a*, matrix *Q*_*a*_ is an incidence matrix relating animals in vector *a* to unknown parent groups in vector *g*_*a*_. Vectors *a* and *e* have means 0 and variances A$\sigma_{a}^{2}$ and $\sigma_{e}^{2}$, respectively.

The corresponding mixed-model equations were:

$$\left[\begin{array}{ccc} X^{\prime }X & X^{\prime }Z & X^{\prime }ZQ\\ Z^{\prime }X & Z^{\prime }Z+A^{-}\alpha & Z^{\prime }ZQ\\ Q^{\prime }Z^{\prime }X & Q^{\prime }Z^{\prime }Z & Q^{\prime }Z^{\prime }ZQ \end{array}\right]\left[\begin{array}{c} \hat{b}\\ \hat{a}\\ \hat{g} \end{array}\right]=\left[\begin{array}{c} X^{\prime }y\\ Z^{\prime }y\\ Q^{\prime }Z^{\prime }y \end{array}\right]$$

Solving the equations the breeding value of an animal *m* will be:

$$a_{m^{*}}=Q\hat{g}+{\hat{a}}_{m}$$

The accuracy of EBV was calculated as recommended by Aguilar et al. (2020):

$${\text{Accuracy}}_{\mathrm{ij}}=1-\frac{{\text{SE}}^{2}}{\left(1+\mathrm{fx}\right)v_{a}}$$

where SE is the standard error for the animal solution *i* in trait *j*, *fx* corresponds to individual inbreeding and *v*_*a*_ is the additive variance $\sigma_{a}^{2}$.

### Comparison of Analysis

Results from different scenarios were compared based on descriptive statistics (i.e., mean and standard errors) of VC, Pearson’s correlations between EBVs grouped by animal status (i.e., bulls with at least 10 daughters, buffalo cows with or without progeny), re-rankings of first 10 bulls, efficiency of selection (SEf) as defined later and genetic trends, estimated by the linear regression of EBVs on year of birth.

The SEf was calculated as proposed by Petrini et al. (2015) and Peškovičová et al. (2004), which defined SEf as the ratio between EBVs excluding (${\bar{x}}_{gg\_GG})$ and including genetic groups $\left({\bar{x}}_{GG\_GG}\right)$:

$$\text{SEf}\left(\%\right)=100\times {\bar{x}}_{gg\_GG}/{\bar{x}}_{GG\_GG}$$

The SEf was calculated for the best 10, 30, and 50% animals, respectively.

### Softwares

Data preparation and editing, and all statistical analysis were performed using the R programming environment v.3.6.1 (R Core Team, 2019), except VC which were estimated using AIREMLF90 (Misztal et al., 2002) and EBV which were obtained using BLUPF90 (Misztal et al., 2018). The R package *optiSel* (Wellmann, 2019) was used to calculate the pedigree-based additive relationship matrix and the package *stats* for the hierarchical cluster analysis (R Core Team, 2019). The analyses were run on the ANASB server^1^ using an Intel^®^ Pentium^®^ CPU G3220 @ 3.00GHz, with 2 CPUs and 16 Gb of RAM.

## Results

### Data Overview

Descriptive statistics for the analyzed traits are shown in Table 1. The deviation from the normal distribution was moderate, with kurtosis values ranging from 0.03 to 2.07. Traits distribution was skewed to the right (Supplementary Figure 2) and the average coefficient of variation was 2.8 and 24.4% for composite and linear traits, respectively.

### Variance Components and Heritability

The VC and heritability estimates from the different scenarios are shown in the Tables 3, 4 for composite and linear traits, respectively. The estimated genetic variance was highest for five linear traits (STAT, FUA, RUW, UD, and TL), intermediate for BD, BL, FA, and TP, while the lowest were for composite traits and BCS. On average, the estimates of the additive variances from the GOLD scenario were the highest, observing largest differences with GC60 for the STAT (−0.60) and FUA (+1.31) traits, respectively.

**TABLE 3:** Component of variance and hereditability for the composite traits obtained in the different pedigree scenario in the IMB.

Scenario	Parameter	FS	ST	FL	UT	YP
GOLD	σ^2^*a*	0.55	0.98	0.74	1.02	0.58
	σ^2^*e*	2.02	2.90	4.67	5.96	2.39
	σ^2^*p*	2.57	3.88	5.41	6.98	2.98
	***h*^2^ ± *s.e.***	**0.22 ± 0.03**	**0.25 ± 0.03**	**0.14 ± 0.03**	**0.15 ± 0.03**	**0.20 ± 0.04**
Y30	σ^2^*a*	0.54	0.89	0.73	0.98	0.55
	σ^2^*e*	2.03	2.98	4.67	6.01	2.43
	σ^2^*p*	2.57	3.87	5.40	6.98	2.97
	***h*^2^ ± *s.e.***	**0.21 ± 0.03**	**0.23 ± 0.03**	**0.14 ± 0.03**	**0.14 ± 0.03**	**0.18 ± 0.04**
Y60	σ^2^*a*	0.55	0.87	0.74	0.99	0.50
	σ^2^*e*	2.02	3.00	4.65	5.99	2.48
	σ^2^*p*	2.56	3.86	5.39	6.98	2.97
	***h*^2^ ± *s.e.***	**0.21 ± 0.04**	**0.22 ± 0.04**	**0.14 ± 0.03**	**0.14 ± 0.03**	**0.17 ± 0.05**
GC30	σ^2^*a*	0.51	0.93	0.78	1.17	0.52
	σ^2^*e*	2.06	2.94	4.62	5.83	2.45
	σ^2^*p*	2.56	3.86	5.40	6.99	2.97
	***h*^2^ ± *s.e.***	**0.20 ± 0.04**	**0.24 ± 0.04**	**0.14 ± 0.03**	**0.17 ± 0.03**	**0.18 ± 0.04**
GC60	σ^2^*a*	0.48	0.83	0.84	1.52	0.51
	σ^2^*e*	2.08	3.02	4.55	5.48	2.46
	σ^2^*p*	2.56	3.85	5.40	7.00	2.97
	***h*^2^ ± *s.e.***	**0.19 ± 0.05**	**0.22 ± 0.05**	**0.16 ± 0.05**	**0.22 ± 0.05**	**0.17 ± 0.06**

*σ^2^a = additive genetic variance; σ^2^e = residual variance; σ^2^p = phenotypic variance; h2 = hereditability; s.e. = standard error.*

**TABLE 4:** Component of variance and hereditability for the linear traits obtained in the different pedigree scenario in the IMB.

Scenario	Parameter	STAT	BD	BL	FA	FUA	RUW	UD	TP	TL	BCS
GOLD	σ^2^*a*	9.33	4.44	4.90	2.89	6.64	6.21	7.69	2.53	10.46	0.030
	σ^2^*e*	17.01	19.19	16.20	28.37	31.34	23.47	22.64	16.62	29.35	0.159
	σ^2^*p*	26.34	23.63	21.10	31.26	37.98	29.68	30.33	19.16	39.81	0.189
	***h*^2^ ± *s.e.***	**0.35 ± 0.03**	**0.19 ± 0.03**	**0.23 ± 0.03**	**0.09 ± 0.02**	**0.17 ± 0.03**	**0.21 ± 0.03**	**0.25 ± 0.03**	**0.13 ± 0.03**	**0.26 ± 0.03**	**0.16 ± 0.03**
Y30	σ^2^*a*	8.82	4.21	4.92	3.14	6.18	6.11	6.76	2.25	10.32	0.025
	σ^2^*e*	17.54	19.32	16.23	28.08	31.67	23.51	23.34	16.88	29.31	0.163
	σ^2^*p*	26.36	23.53	21.15	31.22	37.85	29.62	30.10	19.12	39.63	0.188
	***h*^2^ ± *s.e.***	**0.33 ± 0.03**	**0.18 ± 0.03**	**0.23 ± 0.03**	**0.10 ± 0.03**	**0.16 ± 0.03**	**0.21 ± 0.03**	**0.22 ± 0.03**	**0.12 ± 0.03**	**0.26 ± 0.03**	**0.13 ± 0.03**
Y60	σ^2^*a*	8.50	4.20	4.92	3.14	5.98	5.65	6.63	2.39	10.25	0.026
	σ^2^*e*	17.96	19.26	16.24	28.07	31.75	23.89	23.29	16.71	29.20	0.162
	σ^2^*p*	26.45	23.46	21.15	31.21	37.73	29.55	29.92	19.11	39.45	0.188
	***h*^2^ ± *s.e.***	**0.32 ± 0.04**	**0.18 ± 0.04**	**0.23 ± 0.04**	**0.10 ± 0.03**	**0.16 ± 0.04**	**0.19 ± 0.04**	**0.22 ± 0.04**	**0.13 ± 0.03**	**0.26 ± 0.04**	**0.14 ± 0.04**
GC30	σ^2^*a*	9.10	4.10	4.89	2.99	6.03	5.41	6.97	2.45	9.80	0.028
	σ^2^*e*	17.27	19.40	16.22	28.24	31.77	23.85	23.08	16.66	29.77	0.158
	σ^2^*p*	26.37	23.50	21.12	31.23	37.80	29.26	30.05	19.12	39.57	0.188
	***h*^2^ ± *s.e.***	**0.35 ± 0.04**	**0.17 ± 0.03**	**0.23 ± 0.04**	**0.10 ± 0.03**	**0.16 ± 0.03**	**0.18 ± 0.04**	**0.23 ± 0.04**	**0.13 ± 0.03**	**0.25 ± 0.04**	**0.15 ± 0.04**
GC60	σ^2^*a*	9.93	3.48	5.41	3.09	5.33	5.39	6.61	2.60	9.72	0.026
	σ^2^*e*	16.55	19.95	15.75	28.10	32.38	24.10	23.30	16.51	29.70	0.161
	σ^2^*p*	26.48	23.42	21.16	31.19	37.71	29.49	29.91	19.12	39.43	0.187
	***h*^2^ ± *s.e.***	**0.38 ± 0.05**	**0.15 ± 0.05**	**0.26 ± 0.05**	**0.10 ± 0.04**	**0.14 ± 0.05**	**0.18 ± 0.05**	**0.22 ± 0.05**	**0.14 ± 0.04**	**0.25 ± 0.05**	**0.14 ± 0.05**

*σ^2^a = additive genetic variance; σ^2^e = residual variance; σ^2^p = phenotypic variance; h2 = hereditability; s.e. = standard error.*

**FIGURE 1:**
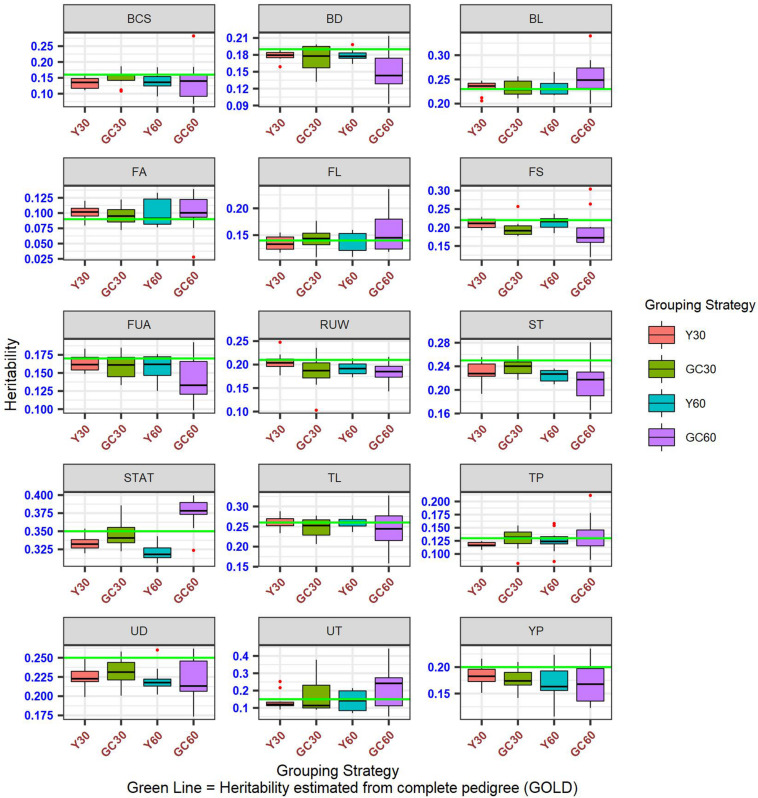
Box plot of the hereditability for composite and linear traits obtained in the different pedigree scenario in the IMB.

Differences among heritability estimates varied according to the trait and the scenario considered and are presented in Figure 1. The green line identifies the heritability from the GOLD scenario. The largest differences were observed in the scenario GC60 for trait UT (0.22 vs. 0.15) and for trait BD (0.15 vs. 0.19). Moreover, GC60 showed the highest within-trait variability, with maximum differences for UT, BCS, and FS (0.39, 0.21, and 0.18, respectively), and minimum differences of 0.08 for RUW and STAT (result not show). Standard errors of heritabilities for all traits were low, ranging from 0.03 (GOLD) to 0.05 (GC60).

### Correlations Between Breeding Values

The correlations between EBVs in the different scenarios are shown in Table 5. Results differed depending on sex: higher estimates were observed in the female population when using a grouping strategy based on the year of birth (Y), while for the bulls higher estimates were observed with the genetic clustering strategy (GC). On average, the correlations were positive and high. Considering buffalo cows with records, the average correlation across traits between official EBVs and EBVs from different scenarios were 0.91, 0.88, 0.84, and 0.79 for Y30, GC30, Y60, and GC60, respectively. The best results were observed for STAT, UD, and TL (average *r* = 0.91) while the most affected trait was FA in the scenario GC60 (*r* = 0.68).

**TABLE 5:** Average correlations for buffalo cows and bulls’ EBVs for the composite and linear traits obtained in the different pedigree scenario in the IMB.

Trait^a^	Female with records				Bulls			
	Y30	Y60	GC30	GC60	Y30	Y60	GC30	GC60
FS	0.92	0.85	0.89	0.80	0.94	0.86	0.93	0.85
ST	0.93	0.87	0.90	0.81	0.89	0.77	0.94	0.84
FL	0.88	0.80	0.88	0.77	0.90	0.70	0.93	0.77
UT	0.89	0.80	0.85	0.73	0.92	0.79	0.92	0.80
YP	0.88	0.78	0.84	0.71	0.91	0.75	0.92	0.85
STAT	0.95	0.89	0.93	0.87	0.95	0.87	0.95	0.89
BD	0.92	0.85	0.89	0.79	0.91	0.77	0.92	0.79
BL	0.92	0.84	0.90	0.81	0.94	0.85	0.95	0.87
FA	0.85	0.75	0.83	0.68	0.76	0.63	0.90	0.77
FUA	0.92	0.85	0.88	0.78	0.90	0.74	0.89	0.76
RUW	0.92	0.86	0.90	0.80	0.92	0.82	0.93	0.81
UD	0.95	0.90	0.92	0.86	0.87	0.74	0.90	0.74
TP	0.88	0.80	0.86	0.75	0.85	0.72	0.91	0.81
TL	0.95	0.90	0.92	0.86	0.88	0.77	0.92	0.77
BCS	0.89	0.82	0.87	0.77	0.76	0.59	0.90	0.78
**Average**	**0.91**	**0.84**	**0.88**	**0.79**	**0.89**	**0.76**	**0.92**	**0.81**

*^a^See Table 1 for trait acronym.*

In the case of bulls, the correlation between EBVs in the grouping GC30 ranged from 0.90 for FUA to 0.96 for STAT and BL, while, in the GC60 scenario the values range between 0.75 for UD to 0.90 for STAT (Table 5). As expected, the highest correlations occurred in scenarios where the proportion of missing pedigree was lower (i.e., Y30 and GC30).

### Accuracy of Breeding Values

**FIGURE 2:**
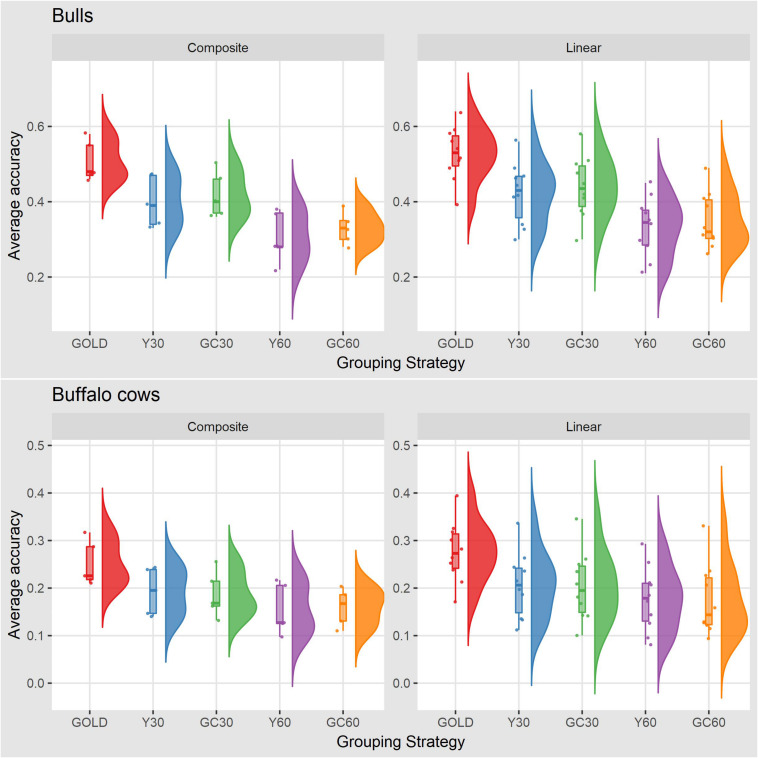
Box plot and histogram of average accuracy for the composite and linear traits by sex obtained in the different genetic group in the IMB.

The accuracy of breeding values across traits and scenarios for bulls with at least 10 daughters and buffalo cows with own record are presented in Table 6. The drop in accuracy for bulls ranged from 0.06 for stature in the scenario GC30 to 0.24 for YP in the scenario Y60. Similar pattern was observed in buffalo cows, with higher accuracies in the Y30 and GC30 scenarios. On average the best results were shown by GC30 (average accuracy = 0.43) and Y30 (average accuracy = 0.42), while the worst results were in the scenario GC60 (average accuracy = 0.34) and Y60 (average accuracy = 0.32) (Figure 2).

**TABLE 6:** Average accuracy buffalo cows and bulls’ EBVs for the composite and linear traits obtained in the different genetic group in the IMB.

Trait^a^	GOLD		Y30		Y60		GC30		GC60	
	Bulls	Female	Bulls	Female	Bulls	Female	Bulls	Female	Bulls	Female
FS	0.55	0.29	0.47	0.24	0.37	0.21	0.46	0.21	0.33	0.17
ST	0.58	0.32	0.47	0.24	0.38	0.22	0.50	0.26	0.39	0.20
FL	0.47	0.22	0.39	0.20	0.28	0.13	0.40	0.16	0.30	0.13
UT	0.48	0.23	0.33	0.14	0.28	0.13	0.37	0.17	0.35	0.19
YP	0.46	0.21	0.34	0.15	0.22	0.10	0.36	0.13	0.28	0.11
STAT	0.64	0.39	0.56	0.34	0.45	0.29	0.58	0.35	0.49	0.33
BD	0.52	0.26	0.42	0.20	0.34	0.17	0.42	0.18	0.31	0.13
BL	0.56	0.30	0.47	0.24	0.38	0.21	0.48	0.23	0.42	0.24
FA	0.39	0.17	0.30	0.11	0.21	0.08	0.3	0.10	0.26	0.09
FUA	0.51	0.25	0.41	0.19	0.30	0.14	0.41	0.17	0.30	0.12
RUW	0.54	0.28	0.44	0.22	0.35	0.18	0.45	0.21	0.33	0.16
UD	0.58	0.32	0.46	0.24	0.37	0.21	0.50	0.25	0.39	0.21
TP	0.46	0.21	0.34	0.14	0.23	0.10	0.37	0.14	0.31	0.13
TL	0.59	0.33	0.49	0.26	0.42	0.25	0.51	0.26	0.41	0.23
BCS	0.49	0.24	0.33	0.13	0.28	0.13	0.38	0.14	0.28	0.11
**Average**	**0.52**	**0.27**	**0.42**	**0.20**	**0.32**	**0.17**	**0.43**	**0.20**	**0.34**	**0.17**

*^a^See Table 1 for trait acronym.*

### Selection Efficiency

The result of the average selection efficiency for the three different selection intensities (top 10, 30, and 50%) for composite and linear trait are summarized in the Table 7. Average of SEf ranged from 22.12 (Top 50 for FL in GC60 scenario) to 85.94% (Top 10 for FS in GC30 scenario) for the composite trait, and from 17.09 (Top 50 for FA in Y60 scenario) to 88.80% (Top 10 for STAT in GC30) for linear traits.

**TABLE 7:** Mean (SD) of efficiency (%) in the selection of the best animals for the composite and linear traits obtained in the different pedigree scenario in the IMB.

Trait^a^	Best	Y30	Y60	GC30	GC60
FS	*10%*	85.11 (4.39)	76.59 (5.96)	85.94 (3.64)	78.64 (5.26)
	*30%*	79.40 (2.77)	67.42 (4.21)	78.47 (2.59)	65.03 (6.70)
	*50%*	58.02 (3.89)	49.24 (5.10)	58.36 (5.12)	47.36 (4.51)
ST	*10%*	85.90 (2.94)	71.76 (5.51)	85.56 (2.31)	74.16 (6.45)
	*30%*	75.29 (3.37)	60.79 (6.84)	76.88 (4.62)	59.54 (4.54)
	*50%*	61.40 (4.61)	47.40 (4.23)	61.42 (5.33)	45.17 (4.57)
FL	*10%*	77.56 (7.12)	52.94 (7.27)	81.86 (4.94)	59.99 (9.58)
	*30%*	65.24 (10.81)	39.40 (8.31)	69.17 (5.18)	39.45 (11.85)
	*50%*	45.27 (12.22)	23.61 (6.33)	51.17 (3.24)	22.12 (8.49)
UT	*10%*	83.86 (4.50)	75.82 (7.74)	82.08 (7.25)	66.32 (9.99)
	*30%*	75.59 (2.78)	59.06 (8.66)	72.53 (5.06)	53.05 (10.68)
	*50%*	55.33 (3.93)	42.65 (8.12)	50.11 (6.21)	36.07 (5.85)
YP	*10%*	81.20 (6.07)	65.42 (9.91)	81.62 (4.73)	73.81 (5.19)
	*30%*	67.99 (6.35)	48.52 (8.86)	61.37 (6.64)	54.01 (8.20)
	*50%*	47.39 (7.15)	32.15 (5.96)	43.55 (4.82)	36.29 (7.72)
STAT	*10%*	88.20 (3.48)	78.17 (4.52)	88.80 (2.79)	75.53 (8.75)
	*30%*	78.07 (2.95)	65.43 (6.32)	77.66 (5.48)	64.95 (5.46)
	*50%*	57.01 (3.31)	45.98 (6.61)	53.91 (4.30)	43.90 (6.23)
BD	*10%*	79.56 (4.03)	65.32 (11.87)	80.48 (4.37)	63.23 (9.87)
	*30%*	66.58 (4.16)	45.51 (12.36)	64.56 (6.59)	45.16 (6.68)
	*50%*	47.75 (3.74)	30.42 (8.87)	42.74 (6.22)	26.65 (5.52)
BL	*10%*	87.32 (5.02)	75.34 (6.01)	87.00 (3.77)	78.22 (5.52)
	*30%*	76.58 (5.90)	63.58 (5.40)	75.95 (4.08)	65.03 (9.02)
	*50%*	51.46 (4.69)	40.51 (5.15)	52.71 (5.65)	43.08 (7.51)
FA	*10%*	72.68 (7.42)	57.76 (10.66)	77.91 (6.54)	58.96 (13.58)
	*30%*	41.43 (6.45)	28.49 (12.88)	58.62 (7.53)	38.32 (12.55)
	*50%*	26.53 (6.16)	17.09 (11.12)	42.98 (7.74)	22.99 (7.66)
FUA	*10%*	78.37 (5.20)	63.29 (9.79)	75.38 (2.78)	66.12 (7.49)
	*30%*	69.73 (6.10)	51.93 (7.88)	67.30 (5.10)	52.37 (8.48)
	*50%*	49.77 (8.29)	35.40 (6.14)	51.03 (2.96)	37.75 (7.66)
RUW	*10%*	78.26 (7.49)	66.44 (7.90)	83.74 (3.74)	68.61 (5.45)
	*30%*	74.16 (7.34)	59.74 (4.37)	74.46 (4.55)	58.60 (4.38)
	*50%*	50.57 (9.91)	38.05 (6.88)	58.88 (4.28)	40.27 (4.11)
UD	*10%*	74.12 (4.78)	61.15 (4.64)	78.28 (5.91)	60.84 (7.90)
	*30%*	62.03 (7.14)	45.55 (5.72)	66.55 (5.47)	45.43 (8.68)
	*50%*	41.64 (4.63)	25.30 (6.08)	47.77 (6.73)	31.05 (5.62)
TP	*10%*	74.87 (4.63)	63.73 (9.03)	79.06 (3.56)	71.11 (9.27)
	*30%*	59.99 (4.50)	43.66 (11.04)	69.87 (4.91)	58.61 (7.78)
	*50%*	40.42 (3.30)	30.73 (9.10)	51.78 (4.33)	41.76 (3.32)
TL	*10%*	76.27 (4.41)	57.14 (7.85)	75.23 (4.48)	57.49 (7.14)
	*30%*	66.10 (3.88)	46.45 (7.27)	66.49 (4.77)	44.98 (7.09)
	*50%*	45.97 (4.30)	29.81 (8.24)	48.94 (4.28)	30.68 (5.25)
BCS	*10%*	58.02 (6.73)	47.45 (8.57)	76.07 (8.29)	58.15 (7.70)
	*30%*	59.16 (5.72)	37.89 (8.57)	67.05 (4.03)	51.64 (7.30)
	*50%*	40.14 (7.26)	23.36 (8.73)	51.47 (5.14)	36.17 (6.06)

*^a^See Table 1 for trait acronym.*

Observing the average intensity of selection across scenarios, the highest value was in GC30 (81.27%), followed by 78.75, 67.41, and 65.22% in Y30, GC60, and Y60, respectively. The average intensity of selections for the best 10, 30, and 50% were 73.16, 60.40, and 42.31%, respectively.

Within each scenario, selection efficiency in composite traits was more effective than in linear traits. When the best 10% of individuals were selected, four out of five composite traits had a selection efficiency higher than 60%, while only three out of 10 linear traits exceeded such a threshold (Table 7). A similar trend was observed selecting 30% (3/5; 4/10 ≥ 50.01%) or 50% (3/5; 4/10 ≥ 32.91%).

In terms of standard deviation, the GC30 scenario showed the lowest standard deviation (average = 4.61), while the values obtained from GC60 and Y60 tend to be higher, with an average SD of 7.94 and 7.82, respectively.

### Re-Ranking

**FIGURE 3:**
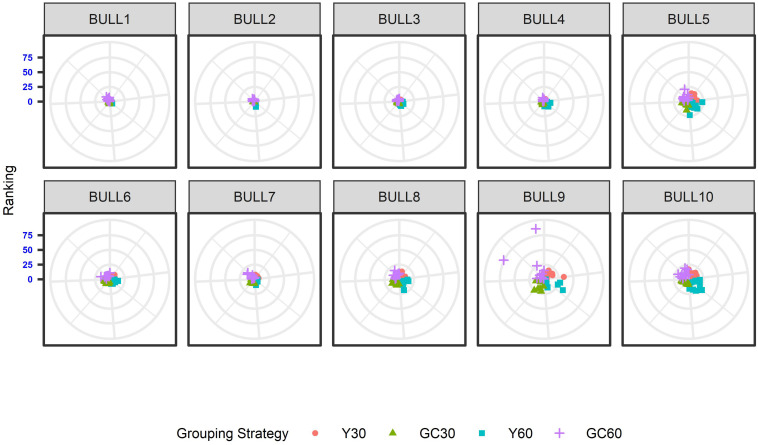
Ten best ranked bulls for the Stature trait according to the different genetic group in the IMB. When the points within the clouds are more dispersed, a larger re-ranking was observed.

**FIGURE 4:**
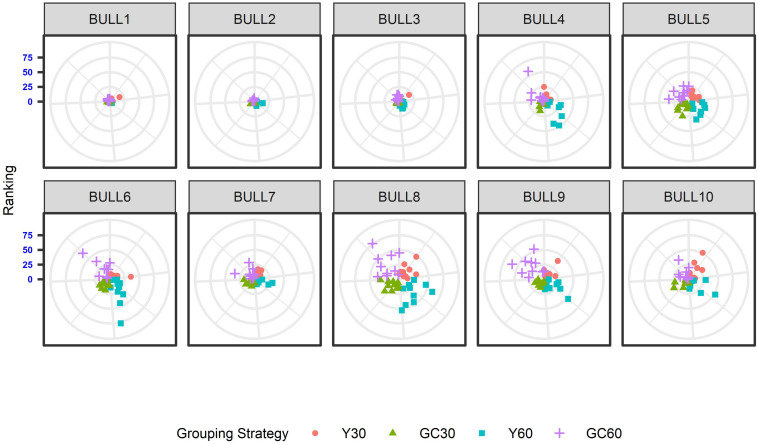
Ten best ranked bulls for the Udder depth trait according to the different genetic group in the IMB. When the points within the clouds are more dispersed, a larger re-ranking was observed.

**FIGURE 5:**
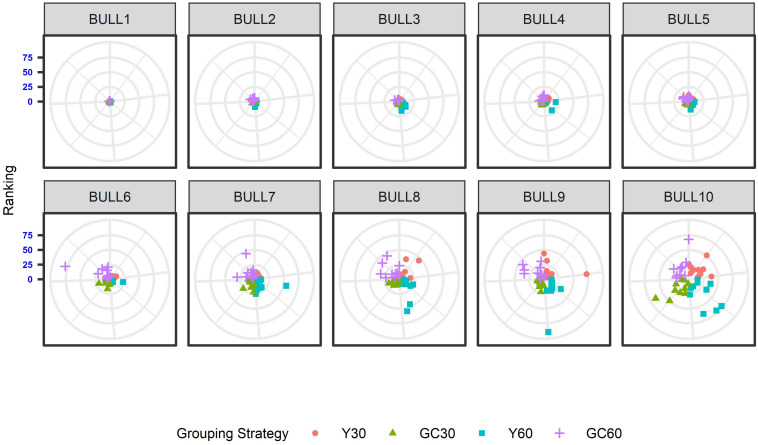
Ten best ranked bulls for the Foot angle trait according to the different genetic group in the IMB. When the points within the clouds are more dispersed, a larger re-ranking was observed.

The effect of the different genetic grouping strategies on the ranking of the bulls was explored using only three linear traits, with high, medium, and low heritability, namely STAT (*h*^2^ = 0.35), UD (*h*^2^ = 0.23), and FA (*h*^2^ = 0.10). Spearman’s rank correlation calculated on 111 bulls in STAT-UD-FA were 0.921–0.884–0.842, 0.913–0.852–0.728, 0.846-0.695-0.659, and 0.811-0.690-0.587 for GC30, Y30, GC60, and Y60, respectively. The consistency of ranking across grouping strategy can also be effectively visualized with a target plot (Biscarini et al., 2016). The rankings of the first 10 bulls across replicates and grouping strategy for STAT, UD, and FA are presented in Figures 3–5, respectively. Each cloud of points represents the ranking of the bull across replicates and within grouping strategy. When the points within the clouds are more dispersed, a larger re-ranking was observed (e.g., BULL9 for STAT trait).

### Genetic Trend

**FIGURE 6:**
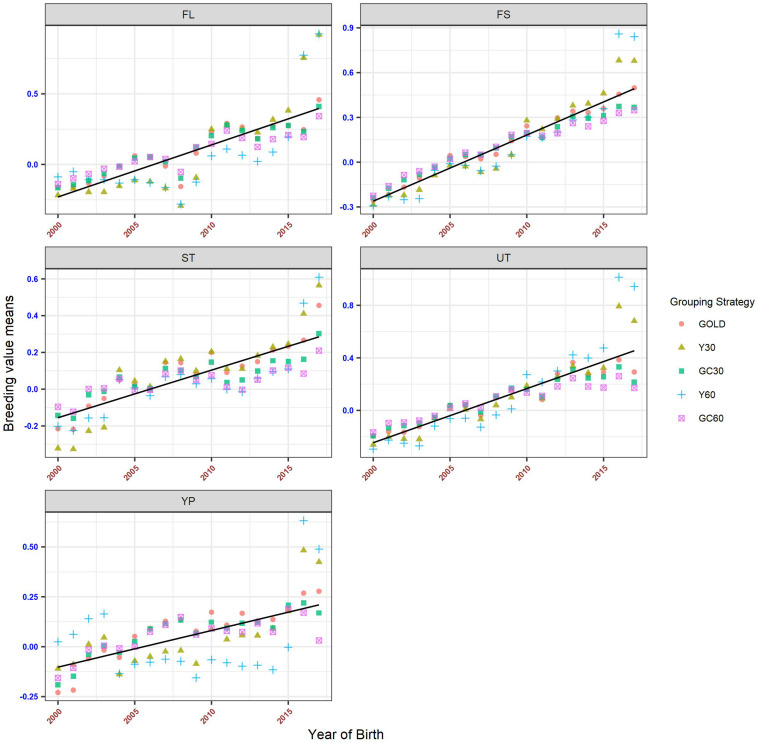
Genetic trend by year of birth for the composite trait, according to the different genetic group.

**FIGURE 7:**
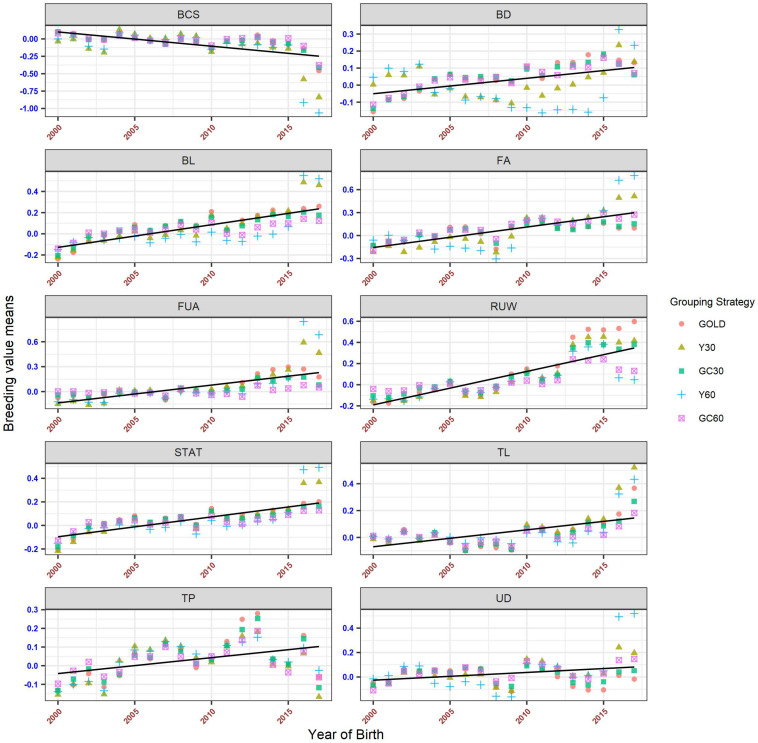
Genetic trend by year of birth for the linear trait, according to the different genetic group.

The genetic trends for both composite and linear traits are presented in Figures 6, 7. Overall a flat trend was observed until year 2013 for all traits. After this year, positive trends were observed and differences among years were enhanced on including genetic groups. For composite traits, an underestimation of the genetic trend was observed when the GC30 and GC60 grouping strategies were used.

Specific behaviors were detected across linear traits. Genetic trends for STAT, FUA, and TL showed the same pattern as the composite traits. BD and BL showed an uneven trend, with a clear positive trend from year 2014. However, when using GC30 and GC60 grouping strategies, EBVs were more regressed than when EBVs were estimated using a grouping strategy based on the year of birth. Similar results were observed for FA and UD where, particularly for recent years, Y30 and Y60 EBVs were higher than GC30 and GC60 EBVs. Finally, BCS showed a flat trend until 2014 followed by a slight decrease, a pattern common to all grouping strategies.

**FIGURE 8:**
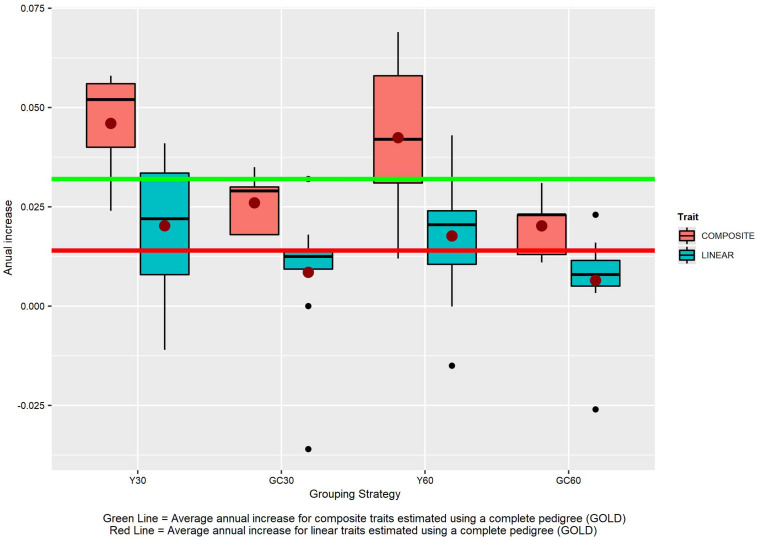
The effect of different grouping strategy on EBVs scale. Average annual increase for the composite and linear trait EBVs, according to the different genetic group.

The different grouping strategies have had an impact on the EBVs scale. From year 2000 the average increase in the scenario without genetic groups (GOLD) was +0.032 for composite traits and +0.014 for linear traits (Figure 8). The average increase in composite traits was +0.046, +0.042, +0.026, and +0.020 when the Y30/Y60/GC30/GC60 genetic group was used, respectively. The same order was observed in the linear trait set with an average increase of +0.020, +0.018, +0.009, and +0.006.

## Discussion

In this study, the effect of two genetic grouping strategies on the estimation of VC and EBV for type traits in a parentage-tested IMB sub-population was evaluated. In the last three years the IMB has experienced an exponential increase in term of registered animals in the Herd Book. As a consequence, IMB is facing a situation where phenotypic data are available for many animals, but some animals lack complete genealogical data. Records from individuals without pedigree information has been excluded from the genetic evaluation or assumed to have an unknown sire. Such practice results in loss of information and potentially could compromise expected genetic gain (Sapp et al., 2007). To mitigate this undesirable effect, several statistical methods have been developed over the years. The use of genetic grouping, parentage probabilities, use of phenotypic information to increase the probability of defining the paternity, iterative empirical Bayesian model (ITER), Bayesian hierarchical model (HIER), and model based on the average relationship matrix (ANRM), have been applied to account for uncertain paternity (Henderson, 1988; Peškovičová et al., 2004; Sapp et al., 2007; Petrini et al., 2015; Carneiro et al., 2017; Shiotsuki et al., 2018; Macedo et al., 2020).

Genetic groups are normally created according to different criteria, for example on the basis of origin, sex, herd, or year of birth of the individual. The creation of the GG is not a simple procedure and can sometimes present some practical problems. Genetic groups modeling must be balanced as groups with few animals might impair the estimation of the GG effect (Rodriguez et al., 1996; Peškovičová et al., 2004; Petrini et al., 2015). At the same time, very large groups are not able to capture the actual differences which exist among individuals. However, (Quaas, 1988) warned about potential bias in defining a determinate grouping strategy due to the effects of confusion between groups. In our case, the “phantom” parents of an individual are always assigned to the same group, because the grouping is based on animal itself, not on its parents, as shown by other studies (Peškovičová et al., 2004; Shiotsuki et al., 2013; Petrini et al., 2015; Wolak and Reid, 2017).

Results have shown that including GG in the mixed model equation had an effect on the estimates of both VC, which can be observed in Tables 3, 4, and EBV (Table 5). Pieramati and Van Vleck (Pieramati and Van Vleck, 1993) obtained lower estimates of additive genetic variance with models that included genetic group. However, we have found that the estimates of VC and EBV with the Y30 and GC30 genetic groups are quite close to the GOLD estimates. These results support the efficiency of the methodology to estimate the true parameters. According to the magnitude of heritability estimates, the GC60 scenario was the one that showed the largest discrepancy with GOLD, confirmed by the highest SE (0.05). Petrini et al. (2015) suggested that such result may be caused by the structure of the group itself. Indeed, the size of GG should be homogeneous and well balanced. In the present study, when a genetic clustering strategy was used, a greater number of groups with a more heterogeneous size was observed. These results depend on the pedigree structure of the IMB, because its completeness is mainly related to the use of artificial insemination. Bulls used for AI have a more complete pedigree both on paternal and maternal side. The fourteen groups used in the GC strategy (Table 2) are based on the relationship matrix and hence are strictly related to the completeness of the paternal line. Indeed in the GC scenario we had a particular group – namely group 1 – which basically included all individuals with no pedigree information and whose size was from 10 to 20-fold larger than the others. Those evidences matched results from Santana et al. (2013) and Shiotsuki et al. (2013) who stressed the importance of the structure of the groups, especially in terms of their number and size (Petrini et al., 2015).

As expected, EBVs accuracy decreased when an increased proportion of missing pedigree was simulated (Table 6). However, when the proportion of missing pedigree was 30%, the average percentage point drop in accuracy was 10 and 7 for bulls and buffalo cows, respectively. We can therefore hypothesize that the contemporary use of the available pedigree information and of the most appropriate GG strategy will mitigate the loss in accuracy of the EBV due to missing pedigree information. Sullivan (1995) suggested the importance of the inclusion of genetic groups in EBV estimation and that data should not be discarded due to the uncertainty of the paternities. Surely, the problem of uncertain paternities might possibly be mitigated by the use of genomic selection (Abdel-Shafy et al., 2020; Macedo et al., 2020; Misztal et al., 2020), however, the genotyping of all animals in a herd might still be too expensive. In the case of IMB, the use of GG is a practical and no cost solution to integrate all the available information into the genetic evaluations process eventually not compromising the accuracy of the results.

On the other hand, Pearson’s correlations between EBVs were generally high in all clustering scenarios. However, Y30 and GC30 scenarios showed the highest correlations. Several studies have shown that correlation coefficients between EBVs lower than 0.70 could suggest changes in the classification of animals (Crews and Franke, 1998; Petrini et al., 2015). Moreover, if we analyze results within traits, we can observe a relationship with heritability value. In our case, the trait that had the lowest correlation coefficient (*r* = 0.68) was FA, whose *h*^2^ was 0.10. In addition, observing the correlations within sex, the Y30/Y60 genetic group strategy showed the highest coefficients for buffalo cows, while for bulls GC30 was the most appropriate for the data. This result was somewhat expected because the strategy based on the hierarchical clustering is strictly related to the relationship matrix, i.e., on the pedigree information. The number of AI bulls in the IMB population is limited (*n* < 100) and most of them have common ancestors. This means that grouping based on the relationship matrix will be possibly biased by the sire’s pedigree. Actually, all individuals with both parents missing have been assigned to group 1 (Table 2), possibly regressing their breeding value. On the other hand, the year of birth has a more balanced behavior and it is less linked to the pedigree. Therefore, our results suggest that the EBV and consequently the ranking of the animals, will be closely influenced by the nature of the trait and by the structure and type of grouping adopted (Shiotsuki et al., 2018).

Considering SEf, several studies suggest that it can be used as a measure of the correlation between the ranking of the best animals obtained in the different analyzes and that would in turn provide information on the degree of efficacy of the genetic grouping strategy (Theron et al., 2002; Peškovičová et al., 2004; Petrini et al., 2015). A value above 70% would indicate that the ranking observed in the different scenarios is stable and does not undergo a significant re-ranking. In relation to what we observed in this study, when the selection intensity is 10%, practically all traits exceeded this threshold (14/15 traits in Y30 and 15/15 in GC30). Meanwhile, in the scenario where the proportion of missing of pedigrees was 60% only 5/15 traits showed a value of SEf higher than 70%. These results suggest that bulls that are above the 90th percentile would experience virtually no important changes in their ranking. Another aspect worth noticing is the standard deviation of SEf. If a large standard deviation is observed, the response to selection will be more unstable and less accurate (Peškovičová et al., 2004). In this regard, the genetic group GC30 showed the lowest standard deviation while results obtained from GC60 and Y60 were more unstable. Consequently, when considering a high correlation and SEf, in addition to a low SD, we retain that the ranking of the bulls will be consistent.

The inclusion of GG in the genetic evaluation could have unpredictable but substantial effects on the estimated genetic trend (Saavedra, 2019). Furthermore, the exclusion of genetic groups or having paternities with “phantom” parents could lead to biased estimates of selection response (Theron et al., 2002). In our study, these expectations are met, observing how the cumulative genetic trends without genetic groups were slightly lower than those estimated with the Y30/Y60 genetic group. Upward trends may indicate that the grouping type “year of birth” may be comparable to those obtained in GOLD. Other study, obtained some indication that the best strategy was grouping phantom sires according to the year of birth and the phantom dams in a single group due to the slow genetic change in females over the generations (Casellas et al., 2007). Theron et al. (2002) and Shiotsuki et al. (2013) observed higher genetic trends when they included GG in the analyses. Those results did not agree with (Petrini et al., 2015) where the inclusion of GG in genetic analyses showed a lower genetic trend.

The effectiveness of including GG on genetic evaluation depends on the genetic structure of the population, the nature of the observed trait (Petrini et al., 2015) and the criterion adopted to define GG. Several authors recommended that the definition of the GG should be a balance between complexity of the method and the adequate representation of genetic differences (Rodriguez et al., 1996; Peškovičová et al., 2004; Petrini et al., 2015; Carneiro et al., 2017; Shiotsuki et al., 2018). The adoption of an inappropriate method may not only have consequences on genetic progress (at the population level), but also on the choice of the best animals that will be used at the herd level. On the other hand, a change in the pedigree structure tends to have a higher impact on traits with medium-low heritability. In our study, this fact occurred with the FA trait, where GC30/GC60 scenarios had the largest correlation with GOLD. On the other hand, for traits with high heritability, the weight of the phenotypic information is high, therefore, the use of GG would have a lesser effect on the estimates. According to Cardoso and Tempelman (Cardoso and Tempelman, 2003), differences between the models that take into account uncertain paternity do not necessarily increase with increasing heritability, but these differences will be greater for the traits of medium-low heritability. In addition, individuals that have a greater number of ancestors or progeny with an incomplete pedigree will be more affected, in particular young animals with no own phenotypic information.

The lack of pedigree information is a common problem among domestic species, being more pronounced in less represented breeds that are mainly managed by small farmers with scarce economic resources. Resolving the uncertainty of paternity has always been a topic of interest to the scientific community and for decades various methodologies have been developed that allow managing the presence of gaps in a relationship matrix. Nowadays, there are different tools to improve the knowledge of genealogical information, such as DNA-based methods, but these are still expensive for breeders. Likewise, in those species that have recently implemented the genetic evaluation system they may face this problem, as they may be in the situation where they possess historical phenotypic data from which it is almost impossible to obtain biological samples due to the absence of a DNA banks.

The prediction of the genetic value with models that consider the uncertainty in paternity have been shown to have better precision (Cardoso and Tempelman, 2003; Sapp et al., 2007; Shiotsuki et al., 2012; Shiotsuki et al., 2013; Carneiro et al., 2017; Shiotsuki et al., 2018). Its effectiveness depends on the definition of the grouping strategy (Petrini et al., 2015), which requires prior knowledge of: (a) the selection process of the breed, (b) the sources of genetic variation present in the population, (c) the intensity of selection or the generational interval. It is clear that GG should be included in the model to improve the accuracy of the EBV of animals with some degree of unknown paternity (Saavedra, 2019). Therefore, the use of genetic groups can be considered an effective alternative in the absence of relationship data for VC and EBV.

## Conclusion

Pedigree completeness is a fundamental requirement of any genetic evaluation. In species other than dairy cattle, the presence of individuals with phenotypic records but with an incomplete pedigree is not a trivial matter. Buffalo breeding is an example of such a situation. We do expect a more extended use of DNA testing which will eventually increase the implementation of genomic selection approaches in Buffalo species as well. However, missing information in the pedigree will still be present and even genomic selection will be faced with the same problem. When a variable proportion of missing pedigree information is present in a population under selection, including genetic groups in the mixed model equations for both VC and EBV estimation is a worth-while and low-demanding approach to mitigate the loss in accuracy. Different strategies can be used to create genetic grouping depending on data distribution across years and on population structure. In the IMB population the best results were obtained when grouping was based on the year of birth. These findings confirmed the possibility of developing a genetic evaluation in populations with uncertain paternities without the need to exclude data or to use only a select of the available population.

## Data Availability Statement

The data analyzed in this study was obtained from Italian National Association of Buffalo Breeders (ANASB). Requests to access these datasets should be directed to d.rossi@anasb.it

## Ethics Statement

Ethical review and approval was not required for the animal study because Animal welfare and use committee approval was not needed for this study as datasets were obtained from pre-existing databases based on routine animal recording procedures.

## Author Contributions

SB and MGC conceived and designed the work. DR, RC, GZ, YG, and DA were responsible for updating and editing the data. SB, RDP, and MGC contributed to analyzing the data and interpreting the results. MGC and SB wrote the manuscript with input from all the authors. All authors revised the manuscript, contributed to the article, and approved the submitted version.

## Conflict of Interest

The authors declare that the research was conducted in the absence of any commercial or financial relationships that could be construed as a potential conflict of interest.

## Footnotes

1 http://www.anasb.it
