# Supplementary material for: Accounting for Genetic Differences Among Unknown Parents in Bubalus bubalis: A Case Study From the Italian Mediterranean Buffalo
Source: Front Genet. 2021 Feb 4;12:625335. doi: 10.3389/fgene.2021.625335 (PMC7901897; doi:10.3389/fgene.2021.625335)
Supplement: Supplementary file 1 [file Data_Sheet_1.docx]

Supplementary Material

## Supplementary Figures

Supplementary Figure 1: Italian Mediterranean Buffalo: grouping based on the genetic distances estimated from the original pedigree. Each rectangle identifies a genetic group (n=14)


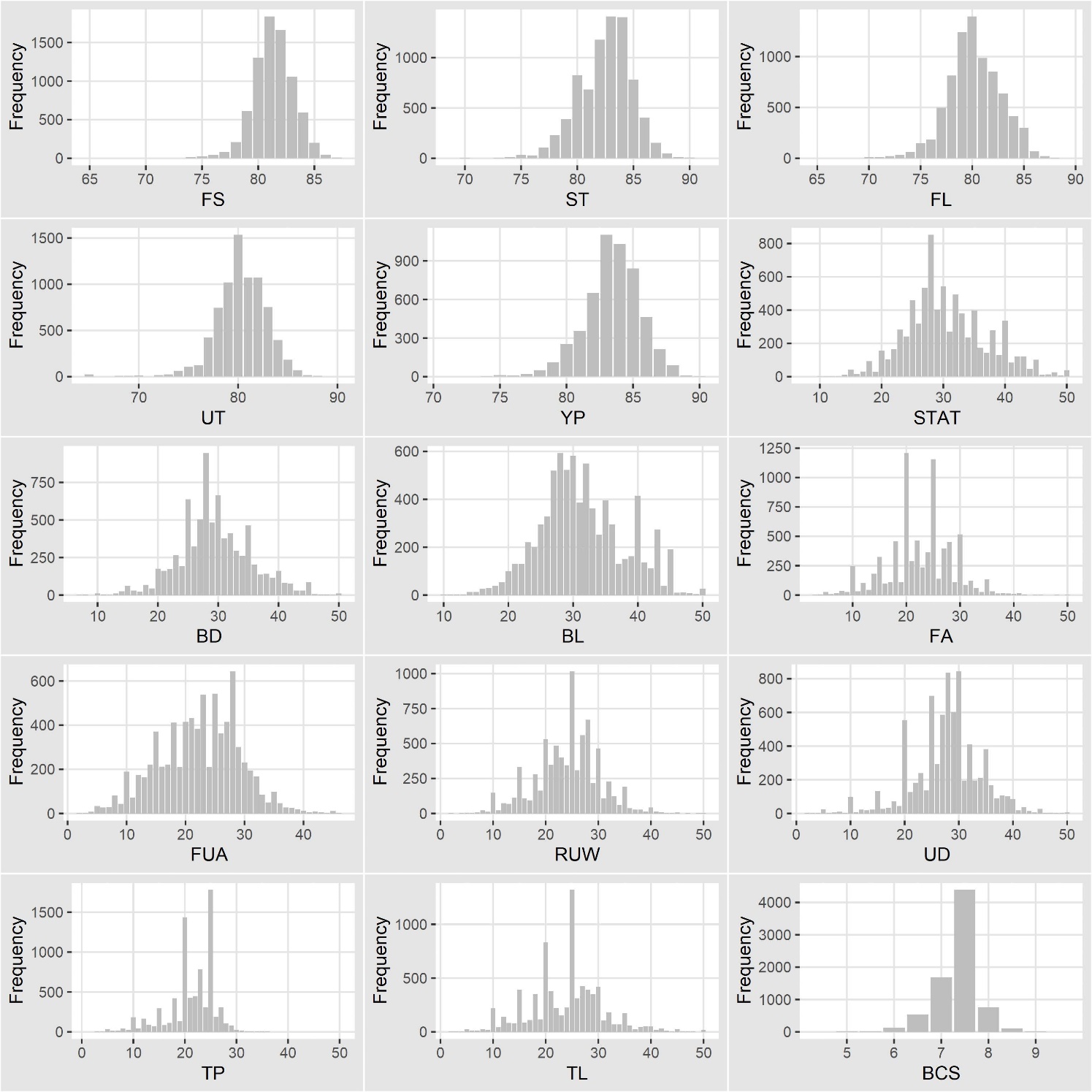


**Supplementary Figure 2.** Composite and linear trait frequency distributions scores in the Italian Mediterranean Buffalo.
